# Supplementary material for: Computational evidence for multi-layer crosstalk between the cadherin-11 and PDGFR pathways
Source: Sci Rep. 2023 Sep 22;13:15804. doi: 10.1038/s41598-023-42624-x (PMC10517159; doi:10.1038/s41598-023-42624-x)
Supplement: Supplementary file 1 — Supplementary Information. [file 41598_2023_42624_MOESM1_ESM.docx]

## **Supplementary material**

**Tables**

**Table S1** Ordinary differential equations governing the activity levels of each protein in the model network.

| Nr. | Equation |
| --- | --- |
| 1 | $\frac{dcdh11}{dt}= \frac{- e^{0.5 \times h} + e^{-h \times(\omega_{cdh11}-0.5)}}{\left( 1-e^{0.5 \times h} \right)\times(1+ e^{-h \times(\omega_{cdh11}-0.5)})} - (k_{i} \times cdh11))$ |
| 2 | $\frac{dpdgfrA}{dt}= \frac{- e^{0.5 \times h} + e^{-h \times(\omega_{pdgfrA}-0.5)}}{\left( 1-e^{0.5 \times h} \right)\times(1+ e^{-h \times(\omega_{pdgfrA}-0.5)})} - (k_{i} \times pdgfrA))$ |
| 3 | $\frac{dpdgfrB}{dt}= \frac{- e^{0.5 \times h} + e^{-h \times(\omega_{pdgfrB}-0.5)}}{\left( 1-e^{0.5 \times h} \right)\times(1+ e^{-h \times(\omega_{pdgfrB}-0.5)})} - (k_{i}\times pdgfrB))$ |
| 4 | $\frac{dras}{dt}= \frac{- e^{0.5 \times h} + e^{-h \times(\omega_{ras}-0.5)}}{\left( 1-e^{0.5 \times h} \right)\times(1+ e^{-h \times(\omega_{ras}-0.5)})} - (k_{i}\times ras))$ |
| 5 | $\frac{draf}{dt}= \frac{- e^{0.5 \times h} + e^{-h \times(\omega_{raf}-0.5)}}{\left( 1-e^{0.5 \times h} \right)\times(1+ e^{-h \times(\omega_{raf}-0.5)})} - (k_{i} \times raf))$ |
| 6 | $\frac{dmek}{dt}= \frac{- e^{0.5 \times h} + e^{-h \times(\omega_{mek}-0.5)}}{\left( 1-e^{0.5 \times h} \right)\times(1+ e^{-h \times(\omega_{mek}-0.5)})} - (k_{i}\times mek))$ |
| 7 | $\frac{derk}{dt}= \frac{- e^{0.5 \times h} + e^{-h \times(\omega_{erk}-0.5)}}{\left( 1-e^{0.5 \times h} \right)\times(1+ e^{-h \times(\omega_{erk}-0.5)})} - (k_{i}\times erk))$ |
| 8 | $\frac{ddusp1}{dt}= \frac{- e^{0.5 \times h} + e^{-h \times\left( \omega_{dusp1}-0.5 \right)}}{\left( 1-e^{0.5 \times h} \right)\times\left( 1+ e^{-h \times\left( \omega_{dusp1}-0.5 \right)} \right)} - (k_{i} \times dusp1))$ |
| 9 | $\frac{dBcat}{dt}= \frac{- e^{0.5 \times h} + e^{-h \times(\omega_{Bcat}-0.5)}}{\left( 1-e^{0.5 \times h} \right)\times(1+ e^{-h \times(\omega_{Bcat}-0.5)})} - (k_{iB} \times Bcat))$ |
| 10 | $\frac{dcyclinD1}{dt}= \frac{- e^{0.5 \times h} + e^{-h \times(\omega_{cyclinD1}-0.5)}}{\left( 1-e^{0.5 \times h} \right)\times(1+ e^{-h \times(\omega_{cyclinD1}-0.5)})} - (k_{i} \times cyclinD1))$ |

**Table S2** Parameter sensitivity analysis for β-catenin initial activity ($Bcat$) versus the inhibition of DUSP1 by β-catenin ($\beta_{{dusp1}_{by_{Bcat}}}$). Steady state activity levels of CyclinD1 and Erk are given for each parameter set in the search space.

|  | Input parameters | | Output activity levels | |
| --- | --- | --- | --- | --- |
| Set A | $\mathrm{Bcat}$ | $\beta_{{dusp1}_{by_{\mathrm{Bcat}}}}$ | $cyclinD1$ | $\mathrm{erk}$ |
| 0 | 0.1 | 0.1 | 0.531146 | 0.408109 |
| 1 | 0.1 | 0.5 | 0.49904 | 0.436102 |
| 2 | 0.1 | 1 | 0.463859 | 0.466805 |
| 3 | 0.1 | 5 | 0.295306 | 0.614448 |
| 4 | 0.1 | 10 | 0.202376 | 0.696361 |
| 5 | 0.3 | 0.1 | 0.531146 | 0.408109 |
| 6 | 0.3 | 0.5 | 0.49904 | 0.436102 |
| 7 | 0.3 | 1 | 0.463859 | 0.466805 |
| 8 | 0.3 | 5 | 0.295306 | 0.614448 |
| 9 | 0.3 | 10 | 0.202376 | 0.696361 |
| 10 | 0.5 | 0.1 | 0.531146 | 0.408109 |
| 11 | 0.5 | 0.5 | 0.49904 | 0.436102 |
| 12 | 0.5 | 1 | 0.463859 | 0.466805 |
| 13 | 0.5 | 5 | 0.295306 | 0.614448 |
| 14 | 0.5 | 10 | 0.202376 | 0.696361 |
| 15 | 0.7 | 0.1 | 0.531146 | 0.408109 |
| 16 | 0.7 | 0.5 | 0.49904 | 0.436102 |
| 17 | 0.7 | 1 | 0.463859 | 0.466805 |
| 18 | 0.7 | 5 | 0.295306 | 0.614448 |
| 19 | 0.7 | 10 | 0.202376 | 0.696361 |
| 20 | 1 | 0.1 | 0.531146 | 0.408109 |
| 21 | 1 | 0.5 | 0.49904 | 0.436102 |
| 22 | 1 | 1 | 0.463859 | 0.466805 |
| 23 | 1 | 5 | 0.295306 | 0.614448 |
| 24 | 1 | 10 | 0.202376 | 0.696361 |

**Table S3**

Parameter sensitivity analysis for activation of DUSP1 by ERK versus inhibition of DUSP1 by β-catenin ($a_{{dusp1}_{by_{erk}}}$ and$\beta_{{dusp1}_{by_{Bcat}}}$). Steady state activity levels of cyclinD1 and Erk are given for each parameter set in the search space.

|  | Input parameters | | Output activity levels | |
| --- | --- | --- | --- | --- |
| Set | $a_{{dusp1}_{by_{\mathrm{erk}}}}$ | $\beta_{{dusp1}_{by_{\mathrm{Bcat}}}}$ | $cyclinD1$ | $\mathrm{erk}$ |
| 0 | 0.1 | 0.1 | 0.465147 | 0.46568 |
| 1 | 0.1 | 0.5 | 0.436817 | 0.490428 |
| 2 | 0.1 | 1 | 0.406142 | 0.51725 |
| 3 | 0.1 | 5 | 0.261913 | 0.643833 |
| 4 | 0.1 | 10 | 0.182202 | 0.714202 |
| 5 | 0.5 | 0.1 | 0.500014 | 0.435253 |
| 6 | 0.5 | 0.5 | 0.469732 | 0.461677 |
| 7 | 0.5 | 1 | 0.436741 | 0.490494 |
| 8 | 0.5 | 5 | 0.279978 | 0.627931 |
| 9 | 0.5 | 10 | 0.193308 | 0.704378 |
| 10 | 1 | 0.1 | 0.531146 | 0.408109 |
| 11 | 1 | 0.5 | 0.49904 | 0.436102 |
| 12 | 1 | 1 | 0.463859 | 0.466805 |
| 13 | 1 | 5 | 0.295306 | 0.614448 |
| 14 | 1 | 10 | 0.202376 | 0.696361 |
| 15 | 5 | 0.1 | 0.634169 | 0.31841 |
| 16 | 5 | 0.5 | 0.59486 | 0.352617 |
| 17 | 5 | 1 | 0.551003 | 0.390807 |
| 18 | 5 | 5 | 0.338844 | 0.57621 |
| 19 | 5 | 10 | 0.226035 | 0.675465 |
| 20 | 10 | 0.1 | 0.677707 | 0.280542 |
| 21 | 10 | 0.5 | 0.63424 | 0.318348 |
| 22 | 10 | 1 | 0.585566 | 0.360707 |
| 23 | 10 | 5 | 0.353034 | 0.563764 |
| 24 | 10 | 10 | 0.232974 | 0.669343 |

**Table S4**

Parameter sensitivity analysis for activation of ERK by MEK versus inhibition of ERK by DUSP1 ($a_{{erk}_{by_{mek}}}$ and$\beta_{{erk}_{by_{dusp1}}}$). Steady state activity levels of cyclinD1 and ERK are given for each parameter set in the search space.

|  | Input parameters | | Output activity levels | |
| --- | --- | --- | --- | --- |
| Set | $a_{\mathrm{erk}_{by_{\mathrm{mek}}}}$ | $\beta_{\mathrm{erk}_{by_{dusp1}}}$ | $cyclinD1$ | $\mathrm{erk}$ |
| 0 | 0.1 | 0.1 | 0.490147 | 0.517636 |
| 1 | 0.1 | 0.5 | 0.46842 | 0.475266 |
| 2 | 0.1 | 1 | 0.446431 | 0.435738 |
| 3 | 0.1 | 5 | 0.351571 | 0.295422 |
| 4 | 0.1 | 10 | 0.296962 | 0.231355 |
| 5 | 0.5 | 0.1 | 0.501117 | 0.540422 |
| 6 | 0.5 | 0.5 | 0.478967 | 0.495397 |
| 7 | 0.5 | 1 | 0.456564 | 0.453561 |
| 8 | 0.5 | 5 | 0.359933 | 0.306161 |
| 9 | 0.5 | 10 | 0.304251 | 0.239347 |
| 10 | 1 | 0.1 | 0.508982 | 0.557383 |
| 11 | 1 | 0.5 | 0.486546 | 0.510368 |
| 12 | 1 | 1 | 0.463859 | 0.466805 |
| 13 | 1 | 5 | 0.366001 | 0.314126 |
| 14 | 1 | 10 | 0.309561 | 0.245271 |
| 15 | 5 | 0.1 | 0.525577 | 0.595009 |
| 16 | 5 | 0.5 | 0.502582 | 0.543541 |
| 17 | 5 | 1 | 0.479341 | 0.496125 |
| 18 | 5 | 5 | 0.379016 | 0.331719 |
| 19 | 5 | 10 | 0.32101 | 0.258347 |
| 20 | 10 | 0.1 | 0.529521 | 0.604342 |
| 21 | 10 | 0.5 | 0.506403 | 0.551763 |
| 22 | 10 | 1 | 0.483039 | 0.503387 |
| 23 | 10 | 5 | 0.382154 | 0.336069 |
| 24 | 10 | 10 | 0.323783 | 0.261577 |

**Table S5**

Parameter sensitivity analysis for activation of cyclin-D1 by DUSP1 versus inhibition of ERK by DUSP1 ($a_{{cyclind1}_{by_{mek}}}$ and$\beta_{{erk}_{by_{dusp1}}}$). Steady state activity levels of cyclinD1 and ERK are given for each parameter set in the search space.

|  | Input parameters | | Output activity levels | |
| --- | --- | --- | --- | --- |
| Set | $a_{{cyclinD1}_{by_{dusp1}}}$ | $\beta_{\mathrm{erk}_{by_{dusp1}}}$ | $cyclinD1$ | $\mathrm{erk}$ |
| 0 | 0.1 | 0.1 | 0.360313 | 0.557383 |
| 1 | 0.1 | 0.5 | 0.339929 | 0.510368 |
| 2 | 0.1 | 1 | 0.319866 | 0.466805 |
| 3 | 0.1 | 5 | 0.239095 | 0.314126 |
| 4 | 0.1 | 10 | 0.19634 | 0.245271 |
| 5 | 0.5 | 0.1 | 0.435932 | 0.557383 |
| 6 | 0.5 | 0.5 | 0.41401 | 0.510368 |
| 7 | 0.5 | 1 | 0.392138 | 0.466805 |
| 8 | 0.5 | 5 | 0.301029 | 0.314126 |
| 9 | 0.5 | 10 | 0.250738 | 0.245271 |
| 10 | 1 | 0.1 | 0.508982 | 0.557383 |
| 11 | 1 | 0.5 | 0.486546 | 0.510368 |
| 12 | 1 | 1 | 0.463859 | 0.466805 |
| 13 | 1 | 5 | 0.366001 | 0.314126 |
| 14 | 1 | 10 | 0.309561 | 0.245271 |
| 15 | 5 | 0.1 | 0.760438 | 0.557383 |
| 16 | 5 | 0.5 | 0.743792 | 0.510368 |
| 17 | 5 | 1 | 0.726151 | 0.466805 |
| 18 | 5 | 5 | 0.639093 | 0.314126 |
| 19 | 5 | 10 | 0.578929 | 0.245271 |
| 20 | 10 | 0.1 | 0.854333 | 0.557383 |
| 21 | 10 | 0.5 | 0.842918 | 0.510368 |
| 22 | 10 | 1 | 0.830608 | 0.466805 |
| 23 | 10 | 5 | 0.766383 | 0.314126 |
| 24 | 10 | 10 | 0.71827 | 0.245271 |

**Figures**


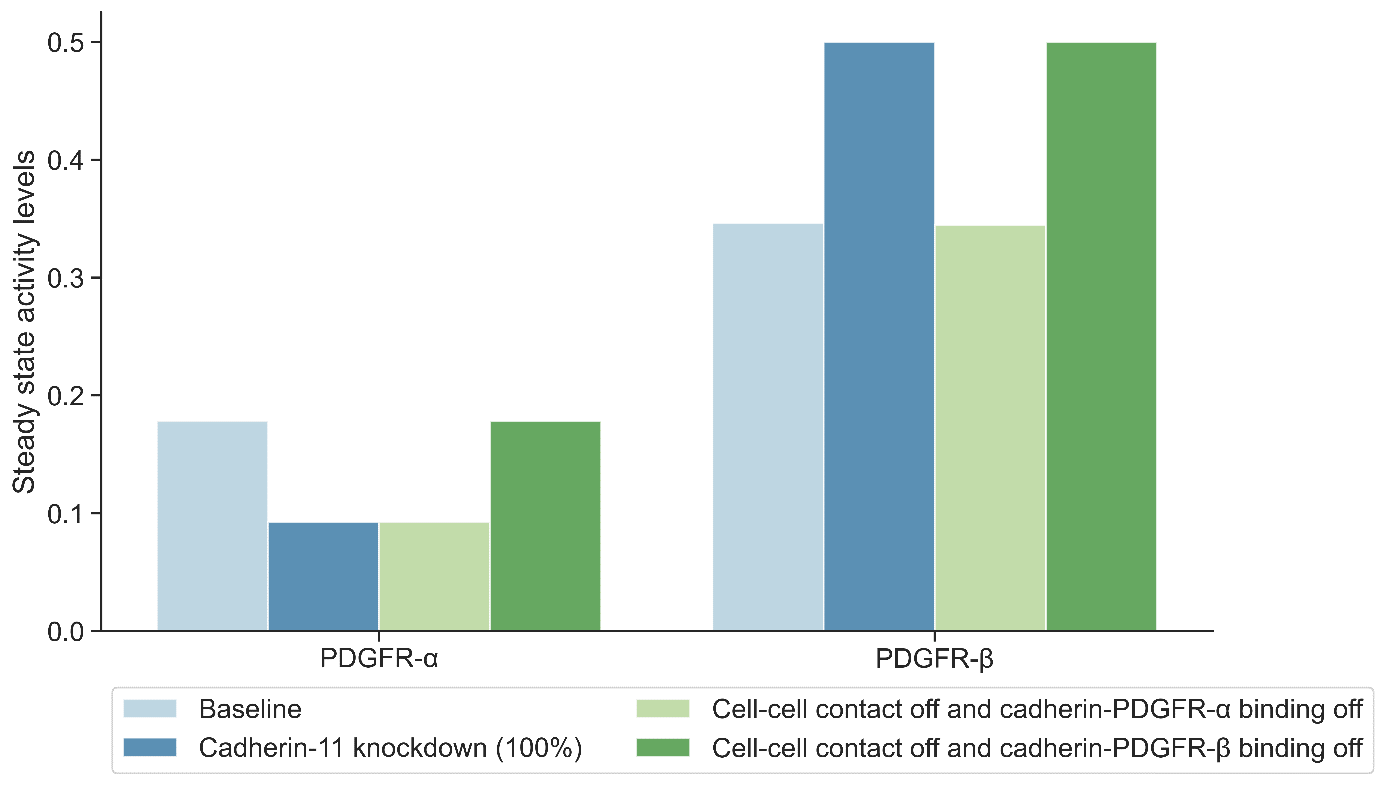


**Figure S1: Model parameters concerning the activity levels of PDGFR-α and PDGFR-β were adjusted to match the previous experimental studies (Madarampalli et al., 2019) and (Passanha et al., 2022)**. In the baseline model, PDGFR-β activity at the steady state is higher than PDGFR-α activity, in accordance with Passanha et al., 2022. Cadherin-11 knockdown simulation resulted in lower than normal PDGFR-α and greater than normal PDGFR-β activity, in accordance with Madarampalli et al., 2019 and Passanha et al., 2022. When cell–cell contact and cadherin-11 binding to PDGFR-α were disabled, lower than normal PDGFR-α activity but no change in the PDGFR-β activity were observed compared to the baseline model. When cell–cell contact and cadherin-11 binding to PDGFR-β were disabled, no change in the PDGFR-α activity level but an increase in PDGFR-β activity were observed compared to the baseline model.


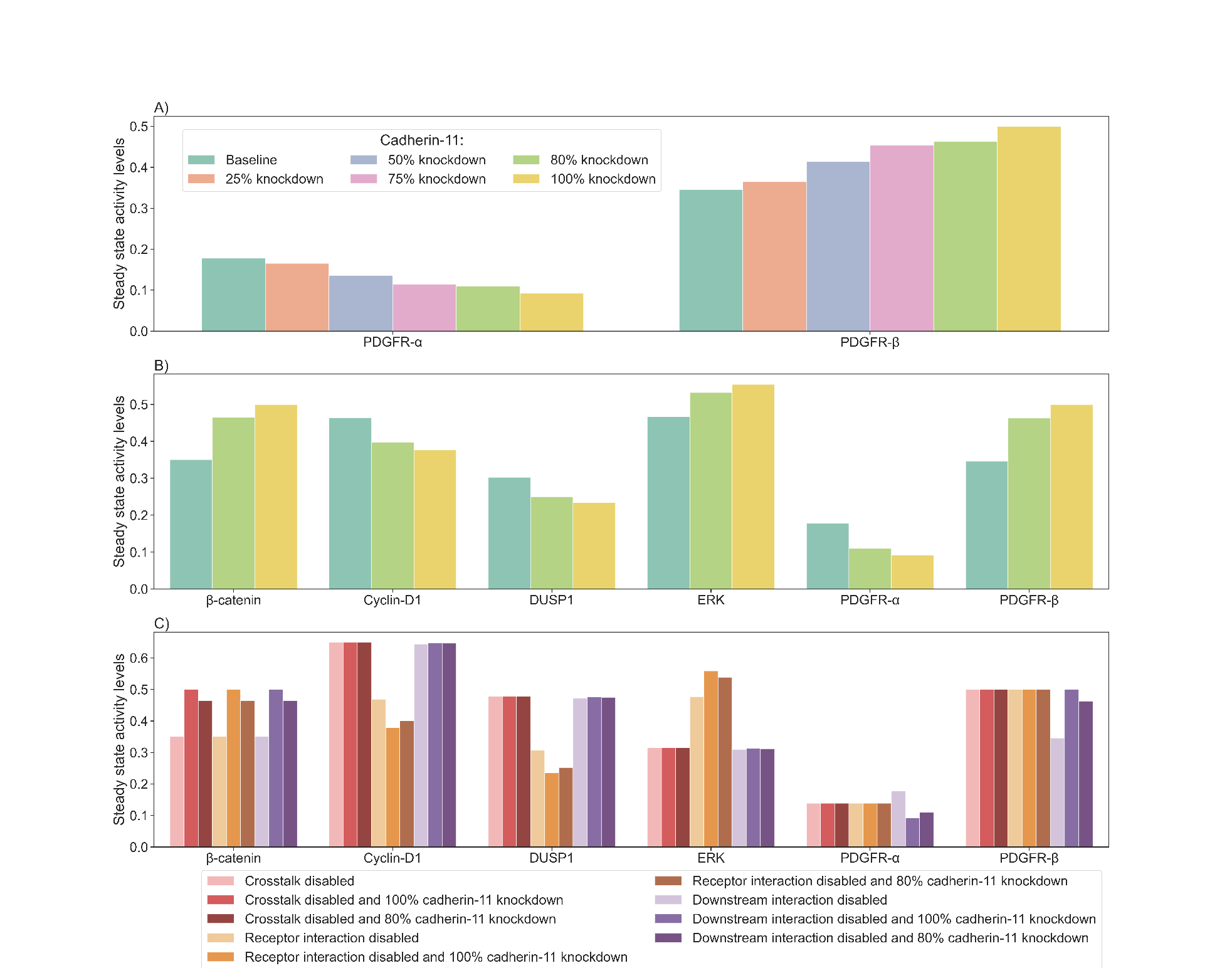


**Figure S2: The effect of knockdown efficiency on the model simulations.** A) The steady state activity levels of PDGFR-α and PDGFR-β change based on the different knockdown efficiencies (25%, 50%, 75%, 80%) compared to the baseline and a full knockdown (100%) as in the main text simulations. We assumed a full loss of cadherin-11 activity in our knockdown simulation and calibrated the model using data from Madarampalli et al., 2019, which did not report their knockdown efficiency and Passanha et al., 2022 which reported a 80% knockdown efficiency. Our test of different knockdown efficiencies showed that there is a monotonic decrease or increase between the baseline and full knockdown for PDGFR-α and PDGFR-β steady state activity levels respectively. As such, since the steady state activity levels of 80% knockdown and 100% knockdown are qualitatively not different, we have chosen the 100% knockdown as our main knockdown simulation and report the results according to a 100% knockdown in the main text. B) The steady state activity levels of β-catenin, cyclin-D1, Dusp1, ERK, PDGFR-α and PDGFR-β are qualitatively similar at 80% knockdown and 100% knockdown, compared to the baseline. C) Steady state activity levels of β-catenin, cyclin-D1, Dusp1, ERK, PDGFR-α and PDGFR-β are qualitatively similar at 80% knockdown and 100% knockdown for crosstalk disabled, receptor interaction disabled and downstram interaction disabled simulations (main text Table 3). For the crosstalk disabled simulation (red bars), the knockdown efficiency does not affect the steady state activity of any proteins in the network except β-catenin because the connection between the RTKs and cadherin-11 as well as the connection between β-catenin and DUSP1 is lost. For the receptor interaction disabled simulation (orange bars), knockdown efficiency does not affect the steady state activity of the RTKs because the interaction between the RTKs and cadherin-11 is lost. Meanwhile the other proteins are minimally affected by the knockdown efficiency, as also shown in panels A and B of this figure. For the downstream interaction disabled simulation (purple bars), knockdown efficiency affects the steady state activity of β-catenin and the two RTKs while other proteins are not affected because the inhibition of DUSP1 by β-catenin is lost in this simulation, which is the most influential level of the multi level crosstalk between cadherin-11 and RTK pathways as explained in the main text.


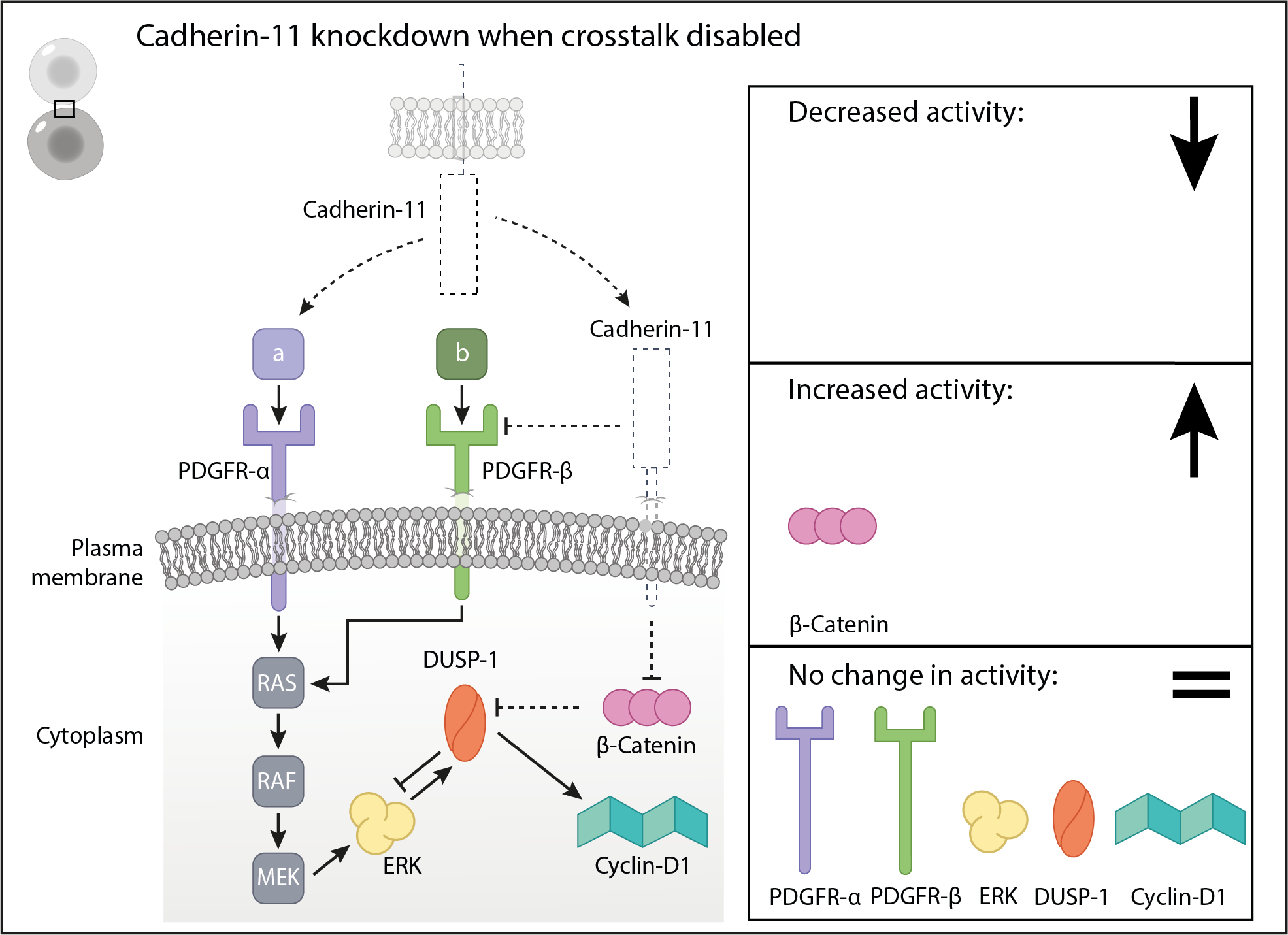


**Figure S3**: **Cadherin 11 knockdown simulation results when the proposed crosstalk is disabled:** The dashed lines indicate where the model components were modified in the simulation setup. No change was observed in the activity levels of growth factor receptors and proliferation-related signaling molecules in case of a cadherin-11 knockdown when the proposed crosstalk mechanisms were disabled.
